# RNA Sequencing Analysis Reveals Transcriptomic Variations in Tobacco (*Nicotiana tabacum*) Leaves Affected by Climate, Soil, and Tillage Factors

**DOI:** 10.3390/ijms15046137

**Published:** 2014-04-11

**Authors:** Bo Lei, Kun Lu, Fuzhang Ding, Kai Zhang, Yi Chen, Huina Zhao, Lin Zhang, Zhu Ren, Cunmin Qu, Wenjing Guo, Jing Wang, Wenjie Pan

**Affiliations:** 1 Key Laboratory of Molecular Genetics, China National Tobacco Corporation, Guizhou Academy of Tobacco Science, Longbatan Road 29, Guanshanhu District, Guiyang 550081, China; E-Mails: leibo198109@gmail.com (B.L.); dingfuzhang2014@gmail.com (F.D.); chenyi83829@gmail.com (Y.C.); zhaohn1983@gmail.com (H.Z.); bambooren1211@gmail.com (Z.R.); 2 Engineering Research Center of South Upland Agriculture, College of Agronomy and Biotechnology, Southwest University, Tiansheng Road 2, Beibei, Chongqing 400715, China; E-Mails: kaizhang2013@gmail.com (K.Z.); zhanglin02150215@gmail.com (L.Z.); drqucunmin@swu.edu.cn (C.Q.); guowenjing0725@gmail.com (W.G.); wangjing217324@gmail.com (J.W.)

**Keywords:** climate factors, soil factors, tillage factors, tobacco leaves, transcriptome, RNA-seq

## Abstract

The growth and development of plants are sensitive to their surroundings. Although numerous studies have analyzed plant transcriptomic variation, few have quantified the effect of combinations of factors or identified factor-specific effects. In this study, we performed RNA sequencing (RNA-seq) analysis on tobacco leaves derived from 10 treatment combinations of three groups of ecological factors, *i.e.*, climate factors (CFs), soil factors (SFs), and tillage factors (TFs). We detected 4980, 2916, and 1605 differentially expressed genes (DEGs) that were affected by CFs, SFs, and TFs, which included 2703, 768, and 507 specific and 703 common DEGs (simultaneously regulated by CFs, SFs, and TFs), respectively. GO and KEGG enrichment analyses showed that genes involved in abiotic stress responses and secondary metabolic pathways were overrepresented in the common and CF-specific DEGs. In addition, we noted enrichment in CF-specific DEGs related to the circadian rhythm, SF-specific DEGs involved in mineral nutrient absorption and transport, and SF- and TF-specific DEGs associated with photosynthesis. Based on these results, we propose a model that explains how plants adapt to various ecological factors at the transcriptomic level. Additionally, the identified DEGs lay the foundation for future investigations of stress resistance, circadian rhythm and photosynthesis in tobacco.

## 1. Introduction

Given that plants root at the same spot throughout their life and that they have large surface areas in contact with the environment, environmental changes have a greater impact on the growth and survival of plants than they do on animals. Several environmental factors, including terrain, climate, soil properties, and soil water have been recognized as affecting plant growth and development and these factors are typically considered when selecting cultivation sites [1]. Among these environmental factors, climate factors (CFs: including light, temperature, air, rainfall, and wind) have the greatest impact on the spatial distribution of vegetation and the yield performance of crops [2], and account for much of the regional variation in crop production. SFs also have a critical effect on plant growth and crop yield, as they determine the physical environment of crop roots and are the major source of nutrients [3]. Soil fertility, texture, organic-matter content, and mineralogy greatly affect the quality and yield of crops. Because SFs can be carefully managed to promote the production of high-yielding and high-quality crops, soil fertility and plant nutrition supplies have been the focus of much research [4,5]. Over 65% of all cropland is supplemented with commercial fertilizer, lime, and soil conditioners, and almost all maize (*Zea may*) and over 80% of wheat (*Triticum aestivum*) and cotton (*Gossypium hirsutum*) are supplemented with commercial nutritional products to improve soil fertility and, ultimately, crop yield in the United States [1]. In addition to environmental factors, TFs also influence the yield and quality of food crops by affecting soil moisture, nutrient availability, temperature, and aeration [6]. Although no-tillage (NT) management has become more popular in North America, as this approach reduces soil erosion and cost and improves soil health, conventional tillage (CT) is still the major farming practice in Asia, South America, and Africa, since the NT approach tends to decrease yield and protein content of crops [7,8]. It is well known that various environmental factors and TFs mutually affect each other [9]. While the effect of each of the above-mentioned factors on crop production has been widely investigated, only a few studies have analyzed the influence of combinations of these factors or identified the specific impact of each factor at the transcriptomic level. Both in tobacco and *Arabidopsis thaliana*, the molecular and metabolic response of plants to a combination of drought and heat stress is distinct from that of plants subjected to each of these stresses applied individually, 454 transcripts in *Arabidopsis* were observed specifically expressed in cells during a combination of drought and heat stress [10,11]. Investigation on phenotypic plasticity of grapevine (*Vitis vinifera*) by comparing the berry transcriptome also revealed the relationships among differential gene expression profiles, environments, growing conditions and ripening parameters and identified several putative candidate genes for the definition of berry quality traits [12].

Recent advances in high-throughput sequencing technology have led to a dramatic increase in the production of sequencing data for RNA-seq and ChIP-seq analyses [13], providing rapid and cost-effective tools to monitor transcriptomic changes. Several studies have assessed global gene expression in different tissues, at different developmental stages, and in response to various environmental stimuli [14,15]. However, the emphasis has always been placed on transcriptomic variation in response to an individual treatment, and the effects of various combinations of treatments have been largely neglected. A transcriptome comparison of fertilized ovary and basal leaf meristem tissue of drought-treated and well-watered maize revealed that more drought-responsive genes were activated in the ovary than in the leaf meristem upon exposure to drought stress [14]. Whole-genome expression profile analysis of lupin (*Lupinus* spp.) identified 2128 genes that were differentially expressed in response to phosphate (Pi) deficiency stress, suggesting that novel mechanisms of Pi deficiency-induced metabolism and cytokinin and gibberellic acid signaling exist [16]. Transcriptome assembly from RNA-seq data identified 28,335 unique genes from sorghum (*Sorghum bicolor* L. Moench) root and shoot tissues challenged with polyethylene glycol (PEG)-induced osmotic stress and exogenous abscisic acid (ABA) [16]. Recent transcriptomic data from the leaves of field-grown rice (*Oryza sativa*) plants at various seasonal, diurnal, and developmental time points along with the corresponding meteorological data were successfully used to develop a statistical model that predicts the influence of variable environmental conditions on transcriptome dynamics, which was found to be predominantly governed by endogenous diurnal rhythms, ambient temperature, plant age, and solar radiation [17]. Model testing on the following year’s rice plants proved that this model was highly accurate at associating gene expression changes with environmental influences, thus suggesting a promising means of translating large amounts of laboratory-learnt knowledge into practical solutions for problems encountered in agricultural production. Despite numerous studies of plant genomes and transcriptomes, few studies have addressed the influence of combinations of environmental and other factors on gene expression profiles. Moreover, few extensive studies have quantified the transcriptome-wide effect of a specific factor although some research has focused on the transcriptomic variation caused by single factors.

Tobacco (*Nicotiana tabacum*) belongs to the agriculturally important Solanaceae family and is a valuable industrial and commercial crop in many countries, although consumption has steadily declined due to increased public awareness of smoking-related health risks and government regulations [18]. It is also an important model organism in plant genetics research, and is ideal for studies in phenotypic diversity, hybridization and ploidy manipulations, and functional characterization. Growth, flowering, and metabolism of tobacco plants are remarkably sensitive to environmental changes, especially to changes in the physical and chemical properties of the soil. Nitrogen and potassium are the nutrient elements with the greatest impact on tobacco growth and development. Optimal levels of nitrogen in the soil increase crop yield, while potassium improves tobacco quality [19]. Compared with CT, minimum tillage (MT) did not have a pronounced effect on tobacco yield, but significantly prolonged the vegetative growth stage [20]. To investigate the transcriptomic variation caused by CFs, SFs, and/or TFs, we analyzed the leaves of tobacco plants subjected to various treatment combinations. Our study provides novel insight into the molecular mechanisms whereby plants adapt to ecological changes and the relationship between various ecological factors at the transcriptomic level.

## 2. Results and Discussion

### 2.1. RNA-Seq Data Analyses

To compare transcriptomic variations in the leaves of tobacco plants exposed to different CFs, SFs, and TFs, ten samples cultivated in Kaiyang County (KY), Weining County (WN), and Tianzhu County (TZ) and exposed to different treatments were collected and used for RNA-seq analysis.

After removal of low quality and contaminated reads, a total of 58,466,453 50 bp raw reads were acquired, ranging from 5.2 to 6.2 M reads per sample, containing 8.28 gigabases (Gb) of sequence data (Table 1). We aligned the sequence reads against the tobacco SGN Unigene database (containing 84,602 unique ESTs) in the Solanaceae Genomics Network (SGN) [21], using TopHat with default parameters [22]. Seventy percent of the total reads were successfully mapped to the reference sequence (Table 1), resulting in about a 50-fold average coverage of the tobacco SGN Unigenes. Previous study demonstrated that 10 and 30 M (75 bp) reads could detect about 80% and all annotated chicken genes, respectively [23]. But, another study also indicated that RNA-seq density generated by about 6 M (36 bp) reads showed a strong congruence with expression metrics from array intensities (Pearson’s *r* = 0.90–0.91) [24]. Considering tobacco is a tetraploid, the sequencing depth in our study would be enough to identify highly expressed DEGs, but might be insufficient for detection of extremely low expression transcripts.

To quantify transcriptomic variations in our samples, Cufflinks was used to assemble all reads into transcript models [22]. Subsequently, expression levels for all transcripts were calculated in fragments per kilobase of exon model per million mapped reads (FPKM), a length-normalized measure of exonic read density that allows expression levels to be compared within or between different samples [25]. Using a threshold of mean FPKM higher than 10, an aggregate of 23,442 to 26,935 mRNA transcripts for each sample was observed. A total of 30,688 unique transcripts were expressed in ten samples (Table S1). The transcripts generated in our study most likely represent almost the complete transcriptome of tobacco leaves. Our results are in accordance with those from a previous tobacco transcriptome analysis in which the transcripts were assembled into a set of 40,642 high-quality unigenes [26]. The transcript number in our study was also much lower than that of the tobacco SGN Unigenes (84,602), indicating that more than half of the SGN unigenes are not expressed in tobacco leaves.

Expression distribution and box plot analysis revealed that ten samples in our study possessed similar expression patterns, with more than 80% of the genes being expressed between 10 and 100 FPKM and around 15% between 100 and 1000 FPKM (Figures S1 and S2). After normalization, box plot and gene expression level distributions both showed that the transcripts of ten samples had similar expression patterns and variation ranges and a normal distribution, suggesting that the ten sets of sequencing data are comparable and suitable for downstream transcriptomic variation analysis.

**Figure 1.**
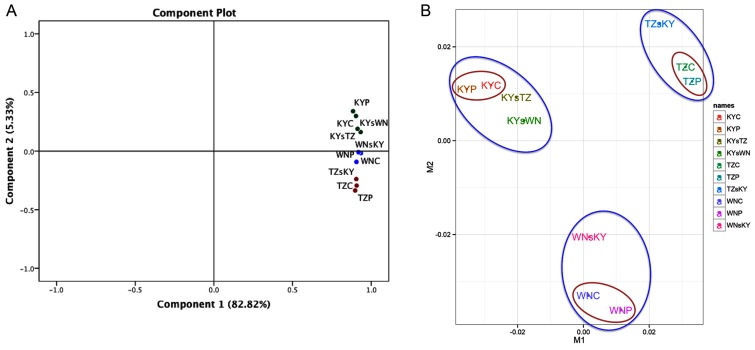
PCA and MDC plots of log_2_-normalized FPKM of ten RNA-seq samples. In the PCA plot (**A**); green, blue, and brown discs represent samples from KY, WN, and TZ, respectively; In the MDS plot (**B**), the brown and blue ellipses indicate transcriptomic variation affected by TFs and CFs, respectively.

To confirm our RNA-seq data and to conduct a preliminarily comparison of the effects of three different ecological factors on tobacco leaf transcriptomes, we implemented a principal component analysis (PCA) of the sample correlation matrix calculated from log_2_-transfromed FPKM values (Figure 1A). The first principal component accounted for 82.82% of the total variability, which in this case corresponds to the reference sequence-specific variance, and the subsequent principal components accounted for 5.33% and 3.35% of overall variance, highlighting the difference between the samples affected by CFs and SFs, respectively (Figure 1 and Table S2), as the values in the component matrix between WN, TZ, and KY were quite different, while those within the same cultivated region varied only slightly (Table 2). Based on the PCA and MDC plots, the largest variance was caused by CFs, and the smallest by TFs (Figure 1). Although it is challenging to make direct comparisons between factors in the above-mentioned two plots, transcriptomic variation caused by SFs is markedly higher than that caused by TFs.

### 2.2. General Trend of DEGs in Tobacco Leaves

To determine the general trend of differentially expressed genes (DEGs) in tobacco leaves exposed to different ecological factors, we identified and analyzed DEGs between the 10 samples in our study (Figure S3 and Table 2). Both up- and down-regulated DEGs were represented with red dots. The number of DEGs between RNA-seq samples retrieved from the same cultivated region was far smaller than those from different cultivated regions, which is in accordance with our PCA and MDC results.

**Figure 2.**
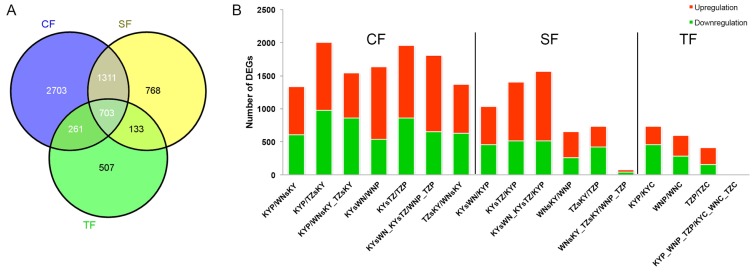
DEGs identified in three treatment combinations. (**A**) A Venn diagram was generated to identify CF-, SF-, and TF-specific DEGs and common DEGs within treatment groups (i), (iii), and (v); (**B**) Number of DEGs in 17 comparison combinations, including seven CF, six SF, and four TF combinations. In each combination, samples were taken from the treatment group with only one different group of ecological factors (CFs, SFs, or TFs). For example, samples of the third comparison combination “KYP/WNsKY_TZsKY” were harvested from the same SFs (KY) and TFs (tillage) and different CFs (KY and sum of WN and TZ). DEGs were determined by comparing the FPKM values of samples before and after slash using the latter as control. An underscore in the sample name indicates an integrated sample. For instance, “WNsKY_TZsKY” represents the average of the sum of WNsKY and TZsKY.

To compare the effect of CFs, SFs, and TFs on the transcriptomes of tobacco leaves, RNA-seq samples were grouped into six different treatments containing 37 pairs of combinations (Tables 2 and S3), including (i) different CFs and the same SFs and TFs; (ii) the same CFs and different SFs and TFs; (iii) different SFs and the same CFs and TFs; (iv) the same SFs and different CFs and TFs; (v) different TFs and the same CFs and SFs; (vi) different TFs and the same CFs and SFs. Based on the screening standard described in Materials and Methods, we found that 6386 genes with FDR <0.5 were differentially expressed in the treatment groups (i), (iii), and (v) (Figure 2), which differ in terms of a single factor. A Venn diagram was created to identify ecological factor-specific DEGs and common DEGs readily affected by changes in ecological factors. The number of CF-, SF-, and TF-specific DEGs was 2703, 768, and 507, respectively, while there were 703 common DEGs in the three treatment groups. In addition, many DEGs were observed between samples that were treated with the same two groups of ecological factors. For instance, CFs and SFs share 1311 common DEGs, whereas TFs have 261 and 133 common DEGs with CFs and SFs, respectively. For samples in group (i) above, KY and TZ (No. 1 and 5 in Table 2) had the most DEGs, while TZ and WN (No. 7 in Table 2) had the fewest DEGs. In group (iii), the number of DEGs in KY soil (No. 14 to 16 in Table 2) was much higher than that in either WN (No. 17 in Table 2) or TZ (No. 18 in Table 2). In group (v), the number of DEGs in KY, WN, and TZ (No. 26 to 28 in Table 2) sequentially decreased, suggesting that changes in TF had diverse effects on the transcriptomes of tobacco leaves sampled from plants cultivated in different regions, and had the greatest effect on plants grown in KY (Table 2 and Figure 2B).

**Figure 3.**
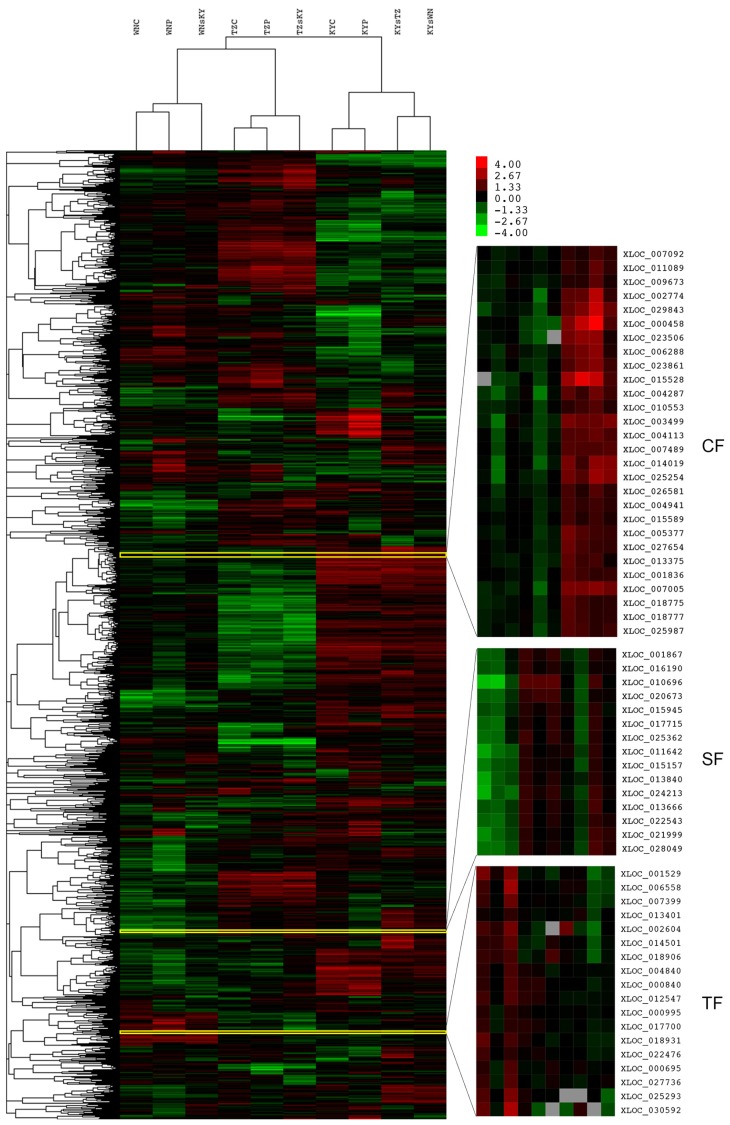
Hierarchical clustering and Treeview visualization of all DEGs. Ten samples from three different cultivated regions with soil exchange and tillage treatments were collected were subjected to RNA-seq, revealing a total of 6,386 DEGs among treatment groups (i), (iii), and (v). Log2 values were used to cluster all the DEGs in Cluster 3.0 using uncentered correlation and the complete linkage method. Results were visualized using Treeview. Left heatmap represents global visualization of all the DEGs, right gene cluster are representative CF-, SF- and TF-specific DEGs. Red indicates genes that are up-regulated, green indicates genes that are down-regulated.

Based on our volcano plot analysis and comparison of number of DEGs affected by different ecological factors (Figure S3), the CFs generally had the greatest effect on transcriptomic variation in tobacco leaves, followed by SFs, and TFs. Cluster analysis of all 6386 DEGs also strongly supported the above conclusion, since expression data of ten samples could be divided into three groups firstly based on three different cultivated regions (CFs), while in the same regions, those between no-tillage and conventional tillage (TFs) were primarily clustered, then grouped with samples derived from soil exchange treatment (Figure 3).

### 2.3. Functional Analysis of Common DEGs Affected by CFs, SFs, and TFs

Genes that are induced by ecological factors play crucial roles in plant growth, development, and adaptation to different ecological stresses. In our study, we identified 703 common DEGs that were simultaneously induced by CFs, SFs, and TFs. These inducible genes might be important for maintaining normal growth and development of tobacco plants and for improving resistance to environmental changes. Based on Gene Ontology (GO) and Kyoto Encyclopedia of Genes and Genomes (KEGG) annotations, 21,410 and 9214 unique genes in our study could be assigned GO and KO terms. Enrichment analysis of common DEGs identified a total of 146 GO and 18 KO terms that were significantly over-represented.

In our GO enrichment analysis (Table S4), 153 genes were found to be involved in response to stimulus (GO:0050896), and 125 of these were annotated as response to stress (GO:0006950). These results suggest that several genes related to the plant’s response to environmental stresses are regulated primarily for adaptation to environmental variation in tobacco plants, which is agreement with our previous study [18]. These stress-responsive genes could be further divided into three classes, *i.e.*, genes that are responsive to a temperature stimulus (GO:0009266, 42 genes), light stimulus (GO:0009416, 29 genes), and oxidative stress (GO:0006979, 32 genes). It is noteworthy that 20 of the 29 genes that respond to a light stimulus are responsive to high intensity light. The first two sets of inducible genes are mainly involved in adaptation to climate change through transcriptional regulation of temperature- and high light-responsive genes in tobacco leaves, whereas the oxidative stress-responsive genes might be important for adaptation to different soils, especially those with different water content. Furthermore, we identified 75 and 30 genes that respond to chemical stimulus (GO:0042221) and hormone stimulus (GO:0009725), suggesting that auxin, ethylene, and abscisic acid (ABA) signaling pathway genes have pivotal roles in the tobacco plant’s response to environmental stresses. Many of the 703 common DEGs had functions related to carbohydrate metabolic processes (GO:0005975, 43 genes) and the cell wall (GO:0005618, 47 genes). These genes have important roles in plant cell wall organization, biosynthesis, and modification, and could increase tobacco plant resistance to abiotic or biotic stresses.

In our KEGG enrichment analysis, KEGG pathway annotations of 203 common DEGs were generated and analyzed for statistical significance (Table S5). Common DEGs were mainly over-represented in ancient, conserved, and secondary metabolic pathways, such as flavonoid biosynthesis (ko00941, 6 genes), phenylpropanoid biosynthesis (ko00940, 12 genes), and starch and sucrose metabolism pathways (ko00500, 12 genes). Although nitrogen metabolism (ko00910, 7 genes) and MAPK signaling pathway (ko04010, 7 genes) genes were also significantly over-enriched in our study, DEGs involved in protein processing in the endoplasmic reticulum (ko04141, 42 genes) were more abundant than those associated with any other pathway. Therefore, based on our GO and KEGG enrichment analyses, we propose that common DEGs affected by CFs, SFs, and TFs are the most important for the survival of tobacco plants under various ecological conditions, particularly stress.

Based on the meteorological data at three cultivated regions (Table S6 [27]), measurement of agronomic traits (Table S7) and activities of antioxidant enzyme superoxide dismutase (SOD, EC 1.15.1.1), peroxidase (POD, EC 1.11.1.7) and catalase (CAT, EC 1.11.1.6) (Table 3), it could be observed that tobacco plant height and number of leaves at 70 days after transplanting (DAT) at cultivation region KY were obviously higher than WN and TZ, and stem perimeter and maximum leaf area at 45 and 70 DAT at cultivation region TZ were smaller than KY and WN. In addition, the influence of soil exchange and tillage treatment on agronomic traits also could be found, for example, four tested agronomic traits between KYC and KYP (TFs), and between KYP and KYsTZ (SFs) were quite different (Table S7). To further investigate tobacco plant growth status the activities of antioxidant enzymes SOD, POD and CAT of tobacco leaves were measured. From Table 3, the impact of environmental variation (CFs) on activities of CAT and POD could be seen. For instance, CAT activities of samples from cultivation region TZ were much higher than KY and WN at 45 DAT, but lower at 70 DAT. Soil exchange (SFs) and tillage treatment (TFs) also showed significant influence on variation of POD activities (Table 3). These results indicated that tobacco plants adapted to variation of CFs, SFs and TFs by adjusting enzyme activity and plant growth, which was in accordance with the above-mentioned GO and KEGG pathway analyses.

### 2.4. Functional Analysis of CF-Specific DEGs

Compared with SFs and TFs, CFs had a much greater influence on the transcriptomic variation of tobacco leaves. In our study, a total of 2703 CF-specific DEGs were identified and found to be over-represented in 64 GO terms and 12 KEGG pathways (Table 4). Among the over-enriched KEGG pathways, plant hormone signal transduction (ko04075), with 35 DEGs, was most enriched (Table S8). Although several genes that respond to hormone stimuli were identified in the above-mentioned common DEGs, these genes differed from hormone-responsive CF-specific DEGs (ko04075). We identified several well-studied hormone-responsive genes amongst the CF-specific DEGs, such as ABA-responsive element binding factor *ABF3* (XLOC_016015) [28], ABA-activated protein kinase (XLOC_003676), jasmonate receptor *CORONATINE INSENSITIVE 1* (*COI1*) [29], auxin-inducible *SAUR* gene (XLOC_018600) [30], and cytokinin receptor histidine kinase *AHK3* (XLOC_014140) [31]. The large number of hormone-responsive genes specifically affected by CFs may improve the adaptive ability of tobacco plants, allowing them to flourish in various growth regions.

DEGs related to the circadian rhythm (ko04712) were present only amongst the CF-specific DEGs. Given the large number of DEGs related to the circadian rhythm (19 in total; Table S9), we conclude that the circadian rhythm has a considerable effect on the transcriptome of tobacco plants. This result is consistent with a recent study that found that the transcriptome of rice leaves was widely affected by three factors: the circadian clock, environmental stimuli, and plant age. Similar to our study on tobacco plants, the entrained circadian clock and temperature had particularly large effects on the transcriptome [32]. We thus suggest that changes in the circadian rhythm cause CF-specific transcriptomic variations in tobacco plants. Among the CF-specific DEGs, we identified a nuclear zinc-finger gene *GIGANTEA* (*GI*, *XLOC_014804*, *XLOC_018429*, *XLOC_021883*, *XLOC_023447*, *XLOC_020085*, *XLOC_015139* and *XLOC_017755*), which governs the diurnal rhythm, promoting plant flowering through the *CONSTANS* (*CO*)–*FLOWERING LOCUS T* (*FT*) regulatory module under long-day conditions [33]. The MYB transcription factors *LATE ELONGATED HYPOCOTYL* (*LHY*, *XLOC_012886* and *XLOC_012888*) and *CIRCADIAN CLOCK ASSOCIATED 1* (*CCA1*) are partially redundant and essential for the maintenance of circadian rhythms in constant light conditions [34], and contribute to plant cold tolerance by regulating the *C-REPEAT BINDING FACTOR* (*CBF*) cold-response pathway [35]. An active transcriptional repressor of *LHY* and *CCA1*, *PSEUDO-RESPONSE REGULATOR5* (*PRR5*, *XLOC_011077*), could directly downregulate *LHY* and *CCA1* expression, forming an interlocking transcriptional-translational feedback loop of the circadian clock in plants [36]. *GI*, *LHY*, and *PRR5* could act as key nodes of the circadian clock regulatory network in the leaves of tobacco plants derived from different cultivated regions, playing pivotal roles in plant growth and development and reflecting the adaptive ability of plants to changing environmental cues.

In addition, genes involved in secondary metabolism pathways (phenylpropanoid biosynthesis, starch and sucrose metabolism) were significantly represented in CF-specific DEGs, although these pathways were also overrepresented in common DEGs. Of the CF-specific DEGs involved in phenylpropanoid biosynthesis, genes encoding 4-coumaroyl-CoA synthase 1 (*4CL1*, *XLOC_024395*), 4-coumaroyl-CoA synthase 3 (*4CL3*, *XLOC_020018* and *XLOC_027613*), cinnamic acid 4-hydroxylase (*C4H*, *XLOC_004844* and *XLOC_024688*), ferulate 5-hydroxylase (*F5H*, XLOC_004677), phenylalanine ammonia-lyase 1 (*PAL1*, *XLOC_010156*), and phenylalanine ammonia-lyase 2 (*PAL2*, XLOC_026730 and XLOC_010164) are involved in lignin biosynthesis, providing the strength necessary for vertical growth and resistance to biotic stresses and plant diseases. These lignin pathway genes often exist in multi-copy, e.g., four copies of *4CLs* and *PALs* were found in the *Arabidopsis* genome. To adapt to climate change and to improve plant disease resistance, various members of these multi-gene families may be activated in tobacco leaves, resulting in increased lignin content and, concomitantly, resistance.

### 2.5. Functional Analysis of SF-Specific DEGs

Soil and nutrients are necessary for the growth and development of tobacco plants in the field. To examine transcriptomic variation in the leaves of plants grown under the same CFs and TFs, but under different SFs, we exchanged soils from the different regions used in this study. Most of the 2915 DEGs attributed to TFs were also differentially expressed in response to changes in CFs and SFs. Only 768 of these DEGs were TF-specific.

KEGG and GO enrichment analyses of all TF-specific DEGs identified 8 and 20 significantly enriched pathways and GO terms, respectively (Tables 5 and S9). Based on these results, DEGs associated with photosynthesis, e.g., antenna proteins (ko00196 and GO:0009523), protein processing in endoplasmic reticulum (ko04141 and GO:0055035), mineral absorption (ko04978 and GO:0000041), and response to stress (GO:0006950) were of special interest. Although stress resistance-related genes were overrepresented in common and CF-specific DEGs, they were also the largest group of SF-specific DEGs. Many SF-specific DEGs were involved in various biotic and abiotic stress responses. Among these genes were *HEAT SHOCK FACTOR 4* (*HSF4*, *XLOC_011884*) and *HEAT SHOCK PROTEIN 70* (*HSP70*, *XLOC_017046* and *XLOC_018164*), which were both strongly induced by heat stress, and *SALT OVERLY SENSITIVE 3* (*SOS3*, *XLOC_008474*), which encodes a calcium sensor that is essential for potassium nutrition and salt tolerance. Proteins encoded by the NB-LRR domain-containing disease resistance gene (*RPPL1*, *XLOC_030092*) might function with other TIR-NBS proteins involved in salicylic acid biosynthesis and systemic acquired resistance. Stress-responsive SF-specific DEGs did not show a significant preference for a specific stress condition, implying that tobacco plants might have evolved the ability to automatically regulate the transcriptome to withstand changes in soil environments.

Other noteworthy GO terms were closely related to mineral nutrient absorption and transport in the root, such as cellular response to phosphate starvation (GO:0016036), high-affinity iron ion transport (GO:0006827), and copper ion transport (GO:0006825), reflecting the potential differences of mineral content between soils from the three cultivated regions. Nitrogen is the most important nutrient element for normal plant growth and development. In our study, common, CF- and TF-specific DEGs associated with nitrogen metabolism were simultaneously overrepresented. This finding suggests that nitrogen in soil plays a crucial role in tobacco plants’ nutrition from the transcriptome-scale perspective. As a component of the complex nucleic acid structure, the significance of phosphorus for plants is second only to nitrogen. SF-specific DEGs, but not common, CF-, and TF-specific DEGs, involved in the response to phosphate (Pi) deficiency were enriched. The differential expression of these genes might be caused by differences in phosphorus nutrient status in soils of the three cultivated regions, as the available Pi in soil at KY was about three and ten folds of that at WN and TZ, respectively (Table S10 [27]). There were several notable Pi-starvation responsive genes in the SF-specific group, such as *SPX* (*SYG1*/*Pho81*/*XPR1*, *XLOC_002815* and *XLOC_000642*), *PHOSPHOLIPASE D P2* (*PLDP2*, *XLOC_021274*), *PLDP1* (XLOC_031030), *SULFO QUINOVOSYLDIACYLGLYCEROL 1* (*SQD1*, *XLOC_012522* and *XLOC_027534*), and *SQD2* (*XLOC_024053* and *XLOC_002768*). In *Arabidopsis thaliana*, repression of *AtSPX3* led to a decrease in tolerance to Pi starvation and enhanced expression of a subset of Pi-responsive genes. Six *SPX* genes in rice were found to have diverse functions in plant tolerance to Pi starvation, five of which were responsive to Pi-deficiency in shoots and/or roots, and involved in the regulation of Pi-signaling network in a complex regulatory system [37]. We propose that differences in Pi concentration and distribution in soils in different cultivated regions might result in adaptive changes of Pi homeostasis during the growth and development of tobacco plants, increasing Pi acquisition and absorption by repressing the expression of *SPX* genes, or *vice versa* (Table S10). Other than nitrogen- and phosphorus-related genes, we identified seven genes involved in copper acquisition and transport, including *copper transporter 1* (*COPT1*, *XLOC_006434* and *XLOC_006435*), *copper transport protein* (*CCH*, *XLOC_006386* and *XLOC_006380*), *copper-transporting ATPase* (*RAN1*, XLOC_000748 and XLOC_000749), and *haloacid dehalogenase*-like *hydrolase family protein* (*PAA2*, XLOC_001489). Among these copper homeostasis-related genes, *COPT1*, *CCH*, and *RAN1* were mainly involved in copper acquisition and transport in leaves, while *PAA2* was mainly responsible for metal ion binding and coupled to transmembrane movement of substances. Since these genes were induced by copper deficiency, ozone, and senescence and are required for copper homeostasis and normal plant growth and development, the available copper concentration in the soils of the cultivated regions WN, TZ and KY were 1.29, 1.53 and 1.87 mg/kg, did not show significant difference. Thus, these DEGs involved in copper homeostasis were highly sensitive to change of copper iron concentration could be assumed.

### 2.6. Functional Analysis of TF-Specific DEGs

To compare the effect of different tillage methods on transcriptomic variation of tobacco leaves, NT and CT approaches were implemented at the three cultivated regions. Overall, TFs had a much smaller effect than CFs and SFs, with a total of 1604 TF-related DEGs being identified, only 507 of which were TF-specific DEGs. KEGG analysis found that TF-specific DEGs involved in photosynthesis (ko00195) showed the highest enrichment level, suggesting that changes in tillage methods would have a significant impact on tobacco plants by altering the transcriptional regulation of photosynthesis genes, especially those related to photosystem I (Tables 6 and S11). More than half of the 13 photosynthesis DEGs encode subunit proteins of the photosystem I reaction center, such as PsaA (XLOC_030806), PasB (XLOC_015064 and XLOC_004737), PasD (XLOC_026534), PasG (XLOC_003245), and PasK (XLOC_014624). Furthermore, TF-specific DEGs encoding ATP synthase B (ATPase B, XLOC_000443), ATPase F (XLOC_001007), ferredoxin (XLOC_007987), and the cytochrome b(6) subunit of the cytochrome b6f complex (PETB, XLOC_007806) and involved in converting light energy into chemical energy, energy absorption, electron transfer, and ATP synthesis, may also contribute to the differences in photosynthetic efficiency of tobacco leaves of plants cultivated by CT or NT. A previous study showed that the photosynthetic rate of rice plants cultivated under NT was significantly higher than that of plants cultivated under CT [38]. This finding is in accordance with the results of our study, which show that TFs affect tobacco plants by modulating the expression of photosynthetic genes.

### 2.7. Validation of RNA-Seq Data by qRT-PCR

**Figure 4.**
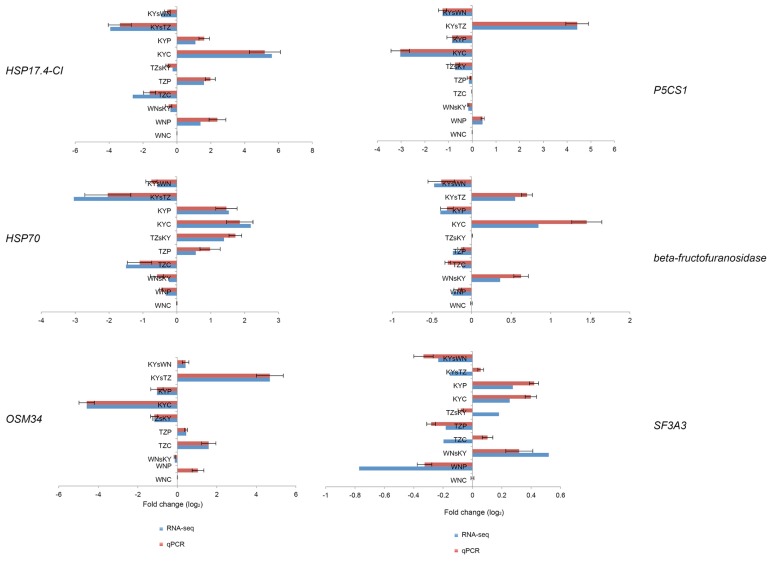
Correlation of differential expression between RNA-seq and qRT-PCR. Five DEGs and one non-DEG were chosen as differentially expressed by RNA-seq. The log_2_-fold change of DEGs obtained from RNA-seq data (blue) *versus* log_2_-fold changes of qRT-PCR derived on the basis of expression levels for treatment (pink) averaged from three samples. All the log_2_-fold changes were calculated using the expression value of WNC as a calibrator. Error bars indicate SD.

To confirm the transcriptome data generated by RNA-seq, qRT-PCR was carried out on five DEGs and one non-DEG randomly selected for their different expression levels. These genes encoded HSP17.4-CI, HSP70, osmotin-like protein (OSM34), delta 1-pyrroline-5-carboxylate synthetase A (P5CS1), beta-fructofuranosidase, and SF3A3 (splicing factor 3A subunit 3), respectively. All genes except for *SF3A3* showed a concordant direction of fold change between RNA-seq and qRT-PCR, using the expression value in WNC to calibrate the data (Figure 4). Although three samples for SF3A3 had different directions of fold change, the remaining seven samples had similar expression patterns. These results confirm the reliability and accuracy of the RNA-seq data in this study.

## 3. Experimental Section

### 3.1. Plant Materials

Tobacco plants (*Nicotiana tabacum* cv. Yunyan 85) used in this study were kindly provided by the Guizhou Tobacco Research Institute, Guiyang, China. Plants were grown in three different cultivated regions of Guizhou Province, including Longgang Town, Kaiyang County (KY), Niupeng Town, Weining County (WN), and Shexue Town, Tianzhu County (TZ) in 2009, as in our previous study [18]. Main meteorological data at different growth stages and basic physiochemical property of soils at three cultivated regions were listed in Tables S7 and S10. To compare the effect of climate factors (CFs; different cultivated regions), SFs (soils of different regions), and TFs (CT or NT) on the transcriptomes of tobacco leaves, soil from KY was exchanged with soil from WN and TZ, and the soil plow layer (soil depth 20–40 cm) at the three regions was broken (*i.e.*, the plough layer (soil depth 0–20 cm) and plow layer were refilled in turn in their original positions after being dug up). In total, ten RNA-seq samples subjected to various treatments were harvested from the three cultivated regions (Table 7). For each treatment, two to three mature leaves (about 65 cm, taken from the middle parts of tobacco plants) were collected from three plants 70 DAT. To minimize the impact of sampling error, sample collection were carried out at 10:00 to 11:00 a.m. for three consecutive sunny days. All samples were immediately frozen in liquid nitrogen and stored at −80 °C.

### 3.2. RNA Preparation

Total RNA was extracted from approximately 100 mg tobacco leaves using a Plant RNA Mini Kit (Watson Biotechnologies, Inc., Shanghai, China), as previously described [39]. RNA was treated with RNase-free DNase I (TaKaRa, Dalian, China) for 30 min at 37 °C to remove all possible DNA contamination. RNA quality was checked by gel electrophoresis (using a 1.2% formaldehyde denaturing agarose gel). RNA concentrations were determined by measuring the absorbance at 260 nm with a NanoDrop ND-2000 Spectrophotometer (Thermo Scientific, Waltham, MA, USA) and an Agilent 2100 BioAnalyzer (Agilent Technologies, Palo Alto, CA, USA). After quantification, RNA samples from each treatment were equivalently pooled for RNA-seq and qRT-PCR analysis.

### 3.3. RNA-Seq Library Preparation and Sequencing

Library preparation and sequencing reactions were conducted in the Beijing Genome Institute (BGI, Shenzhen, China). Briefly, mRNAs were isolated from purified total RNA using magnetic oligo (dT) beads and fragmented, followed by first-strand cDNA synthesis using random hexamer-primed reverse transcription. The second-strand cDNA was generated using buffer, dNTPs, RNase H, and DNA polymerase I. After purification with a QIAquick Gel Extraction Kit (Qiagen, Frankfurt, Gremany), short fragments were resolved for end reparation and adaptor ligation. Following gel electrophoresis, cDNA fragments of approximately 200 bp were isolated and used for cluster generation. Finally, the samples were sequenced using single end (SE) read sequencing with 50 cycles on an Illumina HiSeq 2000, following the manufacturer’s instructions. Base calling was performed with Illumina software Pipeline 1.4 (Illumina, San Diego, CA, USA). RNA-seq data were deposited in the NCBI Sequence Read Archive (SRA) under accession numbers SRR1040764 to SRR1040773.

### 3.4. RNA-Seq Analysis

RNA-seq reads were mapped to 84,602 of tobacco SGN Unigene sequences (ftp://ftp.solgenomics.net/unigene_builds/single_species_assemblies/Nicotiana_tabacum/Nicotiana_tabacum_unigene.v2.seq) retrieved from the Solanaceae Genomics Network (SGN) [21] using TopHat, as described by Trapnell *et al.* [15]. Cufflinks assembled transcripts and quantified transcript abundance in terms of fragments per kilobase of exon per million mapped fragments (FPKM) [22]. Both TopHat and Cufflinks analyses were carried out in default modes. The Cuffdiff program within Cufflinks was used to test for statistically significant differences in transcript expression between 37 comparison pairs (Table 2). Differentially expressed genes (DEGs) were identified using the following two criteria: (i) absolute fold-change >2 and (ii) *q*-value (false discovery rate (FDR)) < 0.05. To further the analysis of our sequencing data, cluster analysis of expression profiles of all DEGs was performed with Cluster 3.0 software with uncentered correlation and complete linkage hierarchical clustering option [40] and the heatmap was visualized using Java TreeView [41].

To better understand the meaning of differential expression, the function of tobacco SGN UniGenes was annotated using BLASTX against the *Arabidopsis thaliana* proteome (version TAIR10 database) with an *e*-value cut-off of 10^−5^ [42,43]. GO annotations of reference SGN UniGenes were performed to retrieve molecular function, biological process, and cellular component terms using Blast2GO (http://www.blast2go.org/) [44]. Enrichment of GO categories among DEGs was assessed by BinGO v2.4.4, a Cytoscape plugin [45,46]. KEGG-based annotation and pathway enrichment analysis was performed using KOBAS 2.0 program [47], which assigned the enzyme commission (EC) numbers and significantly enriched metabolic pathways in DEGs compared with the whole reference sequences [48]. All GO and KEGG statistical tests were corrected for multiple comparisons (Benjamini Hochberg method) [49]. To cluster the samples based on the similarity of gene expression profiles of tobacco leaves, unsupervised principal component analysis (PCA) and multi-dimensional scaling (MDS) were applied. To visualize patterns in different treatments, SPSS 20 (IBM Corp., Armonk, NY, USA) and the R package CummeRbund v2.0.0 were used [50]. The expression of all detected genes in our RNA-seq data was subjected to PCA and MDS analysis and plotted.

### 3.5. Quantitative Real-Time PCR (qRT-PCR) Validation

Five DEGs and one non-DEG identified by RNA-seq were assayed by qRT-PCR. Gene-specific primers were designed based on the nucleotide sequence of the chosen unigenes using Primer 3.0 software [51]. Primers used in this study are summarized in Table S12. The same total RNAs were used as those in the RNA-seq experiments. cDNA synthesis, qRT-PCR cycling conditions, amplification efficiency, and specificity assessment were as described in Lei *et al.* [18]. Briefly, 1 μg of DNaseI-treated total RNA was reverse-transcribed using the PrimeScript RT Master Mix (TaKaRa). qRT-PCR was performed with SYBR Premix Ex Taq II (TaKaRa) using the CFX96 Touch Real-Time PCR Detection System (Bio-Rad, Hercules, CA, USA). Three independent biological replicates were analyzed per sample. The expression level of each sample was calculated using the 2^−ΔΔ^*^C^*^t^ method, with the housekeeping gene *NtEF-1α* serving as an internal control, as its expression is stable in tobacco plants [52,53].

### 3.6. Measurement of Agronomic Traits and SOD, CAT and POD Activities of Tobacco Leaves

To assess the impact of different ecological factors on tobacco plants, four agronomic traits, including plant height, stem perimeter, number of leaves and maximum leaf area of tobacco plants were determined at 45 and 70 DAT. A portable leaf area meter (LI-COR, model LI-3000) was used for measuring the area of the maximum leaf of tobacco plant in each treatment. The activities of SOD, POD and CAT were determined spectrophotometrically. For extraction of SOD, POD and CAT, about 1 g of tobacco leaves samples were ground under liquid nitrogen and homogenized in 10 mL of the extraction buffer containing 50 mM phosphate buffer (pH 7.8) and 1% polyvinylpyrrolidone (PVP). The homogenate was centrifuged at 10,000× *g* at 4 °C for 10 min. The supernatants obtained after centrifugation were used for the enzyme activity analyses. SOD and CAT activities were measured using SOD and CAT Detection Kits (A001 and A007, Nanjing Jiancheng Bioengineering Institute, Nanjing, China) according to the manufacturer’s instructions. Total POD activities were determined by spectrophotometrically monitoring guaiacol oxidation at 470 nm following a previous described method [54]. Values were expressed in units of SOD, POD or CAT activities per gram wet-weight of tissue.

## 4. Conclusions

In conclusion, we performed RNA-seq analysis on tobacco leaves derived from 10 treatment combinations of three ecological factors, CFs, SFs and TFs. RNA-seq analysis generated 58,466,453 reads, which assembled into 30,688 unique transcripts. We detected 4980, 2916, and 1605 DEGs that were affected by CFs, SFs, and TFs, which included 2703, 768, and 507 specific and 703 common DEGs, respectively. Plots of PCA and MDC, and cluster analysis all revealed that the greatest transcriptomic variation was caused by CFs, followed by SFs, with the ratio of factor-specific impacts of CFs:SFs:TFs being about 5:1.5:1. GO and KEGG enrichment analyses showed that genes involved in abiotic stress responses and secondary metabolic pathways were overrepresented in the common and CF-specific DEGs, implying that these genes mediate adaptation to environmental variation. In addition, we noted enrichment in CF-specific DEGs related to the circadian rhythm, SF-specific DEGs involved in mineral nutrient absorption and transport, and SF- and TF-specific DEGs associated with photosynthesis. Based on these results, we propose a model that explains how plants adapt to various ecological factors at the transcriptomic level. Additionally, these data would be useful in selecting candidate genes for future investigations of stress resistance, the circadian rhythm, nutrient absorption, and photosynthesis in tobacco.

## Figures and Tables

**Table 1.** Summary of RNA-seq reads mapping to reference genes.

Sample	Total reads	Mapped reads	% Mapped reads	Number of transcripts
KYC	6,010,205	4,164,387	69.29%	26,864
KYP	6,127,551	4,276,322	69.79%	25,823
KYsWN	5,566,935	3,894,388	69.96%	25,001
KYsTZ	6,020,409	4,162,982	69.15%	25,550
WNC	5,252,135	3,663,806	69.76%	23,971
WNP	5,913,266	4,259,827	72.04%	24,185
WNsKY	5,218,808	3,704,089	70.98%	24,261
TZC	6,091,837	4,254,123	69.83%	25,128
TZP	6,005,103	4,222,155	70.31%	25,540
TZsKY	6,260,204	4,455,821	71.18%	26,100
Overall	58,466,453	41,057,900	70.22%	31,057

KYC, TZC, and WNC represent samples came from KY, TZ, and WN without soil exchange and tillage treatment. KYP, TZP, and WNP represent samples harvested from the corresponding cultivated regions with tillage treatment. KYsTZ and KYsWN represent samples harvested from KY grown in soil from TZ and WN, respectively; TZsKY and WNsKY indicate samples collected from TZ and WN, respectively, and grown on KY soil. Total reads corresponds to the initial output of sequencing reads. Mapped reads refers to the number of reads mapped to the tobacco SGN Unigene reference sequence.

**Table 2.** The number of DEGs in tobacco leaves affected by different CFs, SFs, and/or TFs.

Group	No.	Combination	Number of *DR* genes	Number of *UR* genes	Number of *DEG*s
(i) Different CFs and the same SFs and TFs	1	KYP/WNsKY	610	722	1332
	2	KYP/TZsKY	971	1029	2000
	3	KYP/WNsKY_TZsKY	858	679	1537
	4	KYsWN/WNP	533	1100	1633
	5	KYsTZ/TZP	857	1104	1961
	6	KYsWN_KYsTZ/WNP_TZP	648	1155	1803
	7	TZsKY/WNsKY	631	739	1370

(ii) The same CFs and different SFs and TFs	8	KYsWN/KYC	505	330	835
	9	KYsTZ/KYC	654	585	1239
	10	KYsWN_KYsTZ/KYC	558	604	1162
	11	WNsKY/WNC	164	242	406
	12	TZsKY/TZC	331	513	844
	13	WNsKY_TZsKY/WNC_TZC	8	18	26

(iii) Different SFs and the same CFs and TFs	14	KYsWN/KYP	462	576	1038
	15	KYsTZ/KYP	514	893	1407
	16	KYsWN_KYsTZ/KYP	518	1041	1559
	17	WNsKY/WNP	255	395	650
	18	TZsKY/TZP	416	315	731
	19	WNsKY_TZsKY/WNP_TZP	44	30	74

(iv) The same SFs and different CFs and TFs	20	WNsKY/KYC	960	506	1466
	21	TZsKY/KYC	1168	737	1905
	22	WNsKY_TZsKY/KYC	662	543	1205
	23	KYsWN/WNC	362	850	1212
	24	KYsTZ/TZC	668	1143	1811
	25	KYsWN_KYsTZ/WNC_TZC	317	709	1026

(v) Different TFs and the same CFs and SFs;	26	KYP/KYC	452	277	729
	27	WNP/WNC	289	307	596
	28	TZP/TZC	151	259	410
	29	KYP_WNP_TZP/KYC_WNC_TZC	0	0	0

(vi) The same TFs and different CFs and SFs	30	KYC/WNC	523	1212	1735
	31	KYC/TZC	710	1412	2122
	32	KYC/WNC_TZC	531	794	1325
	33	TZC/WNC	625	821	1446
	34	KYP/WNP	750	1082	1832
	35	KYP/TZP	1034	1109	2143
	36	KYP/WNP_TZP	1144	1065	2209
	37	TZP/WNP	529	956	1485

*UR* genes: up-regulated genes; *DR* genes: down-regulated genes. In each combination, *DEG*s are identified from expression level comparison between samples before and after the slash, using the latter as reference. Underscore between samples indicate that these samples are regarded as an integral whole sample for transcriptomic comparison. For example, the FPKM value of each gene in WNsKY_TZsKY in combination 3 is calculated from samples WNsKY and TZsKY.

**Table 3.** CAT, POD and SOD activities of tobacco leaves.

Location	Treatments	45 DAT			70 DAT		

		CAT	POD	SOD	CAT	POD	SOD
KY	KYC	140.8	1551.3	611.7	101.4	6298.3	481.3
	KYP	128.0	2181.9	648.2	93.5	6914.7	483.9
	KYsTZ	116.4	4275.2	594.0	108.8	8468.2	545.4
	KYsWN	124.5	3929.5	654.1	74.7	9298.9	454.1

TZ	TZC	378.7	3929.3	511.5	94.3	4362.9	500.7
	TZP	391.9	4697.0	476.8	134.3	4474.2	449.5
	TZsKY	361.2	3435.4	514.5	175.9	3724.5	499.4

WN	WNC	175.7	1780.9	509.4	142.4	5180.0	516.9
	WNP	177.7	2255.6	469.8	144.2	6428.5	470.8
	WNsKY	208.3	1426.6	507.7	143.5	4348.7	492.8

Values were expressed in units of enzyme activity per gram wet weight of tissue.

**Table 4.** Over-represented GO terms of CF-specific DEGs.

GO-ID	*p*-value	Input number	Background number	Description
0048578	4.00 × 10^9^	8	8	positive regulation of long-day photoperiodism, flowering
0010378	4.00 × 10^9^	8	8	temperature compensation of the circadian clock
0009813	7.20 × 10^9^	20	52	flavonoid biosynthetic process
0042398	1.51 × 10^8^	44	200	cellular amino acid derivative biosynthetic process
0055114	5.23 × 10^8^	237	1916	oxidation reduction
0010229	1.08 × 10^7^	11	19	inflorescence development
0006575	2.18 × 10^7^	57	316	cellular amino acid derivative metabolic process
0009812	2.20 × 10^7^	20	62	flavonoid metabolic process
0009699	4.81 × 10^7^	23	82	phenylpropanoid biosynthetic process
0048586	5.16 × 10^7^	8	11	regulation of long-day photoperiodism, flowering
0006857	7.65 × 10^7^	17	50	oligopeptide transport
0015833	7.65 × 10^7^	17	50	peptide transport
0009698	9.35 × 10^7^	29	123	phenylpropanoid metabolic process
0019748	1.01 × 10^6^	44	230	secondary metabolic process
0006355	1.57 × 10^6^	161	1262	regulation of transcription, DNA-dependent
0045449	1.99 × 10^6^	161	1267	regulation of transcription
0051252	2.52 × 10^6^	161	1272	regulation of RNA metabolic process
0008215	3.27 × 10^6^	6	7	spermine metabolic process
0006597	3.27 × 10^6^	6	7	spermine biosynthetic process
0010556	6.96 × 10^6^	162	1304	regulation of macromolecule biosynthetic process
0008295	7.34 × 10^6^	8	14	spermidine biosynthetic process
0031326	8.48 × 10^6^	166	1347	regulation of cellular biosynthetic process
0009889	1.05 × 10^5^	166	1352	regulation of biosynthetic process
0008216	1.45 × 10^5^	8	15	spermidine metabolic process
0006835	2.38 × 10^5^	7	12	dicarboxylic acid transport
0051171	2.62 × 10^5^	167	1384	regulation of nitrogen compound metabolic process
0019219	2.89 × 10^5^	165	1367	regulation of nucleobase, nucleoside, nucleotide and nucleic acid metabolic process
0015798	3.15 × 10^5^	5	6	myo-inositol transport
0006596	6.03 × 10^5^	9	22	polyamine biosynthetic process
0006833	6.17 × 10^5^	11	32	water transport
0042044	6.17 × 10^5^	11	32	fluid transport
0006725	9.44 × 10^5^	52	341	cellular aromatic compound metabolic process
0051258	1.06 × 10^4^	13	45	protein polymerization
0006595	1.79 × 10^4^	10	30	polyamine metabolic process
0080090	1.82 × 10^4^	170	1466	regulation of primary metabolic process
0000160	1.91 × 10^4^	25	130	two-component signal transduction system (phosphorelay)
0015791	2.52 × 10^4^	5	8	polyol transport
0015850	2.52 × 10^4^	5	8	organic alcohol transport
0009610	2.95 × 10^4^	4	5	response to symbiotic fungus
0009873	3.36 × 10^4^	13	50	ethylene mediated signaling pathway
0010468	3.41 × 10^4^	162	1405	regulation of gene expression
0019438	3.46 × 10^4^	33	198	aromatic compound biosynthetic process
0006629	3.87 × 10^4^	98	784	lipid metabolic process
0042752	5.76 × 10^4^	8	23	regulation of circadian rhythm
0008610	6.35 × 10^4^	58	422	lipid biosynthetic process
0008202	6.75 × 10^4^	12	47	steroid metabolic process
0051552	7.97 × 10^4^	8	24	flavone metabolic process
0051553	7.97 × 10^4^	8	24	flavone biosynthetic process
0051554	7.97 × 10^4^	8	24	flavonol metabolic process
0051555	7.97 × 10^4^	8	24	flavonol biosynthetic process
0035235	8.07 × 10^4^	6	14	ionotropic glutamate receptor signaling pathway
0007215	8.07 × 10^4^	6	14	glutamate signaling pathway
0060255	8.65 × 10^4^	163	1444	regulation of macromolecule metabolic process
0071369	9.03 × 10^4^	13	55	cellular response to ethylene stimulus
0009409	9.30 × 10^4^	42	286	response to cold
0031323	9.94 × 10^4^	184	1661	regulation of cellular metabolic process
0009755	1.12 × 10^3^	45	315	hormone-mediated signaling pathway
0032870	1.13 × 10^3^	46	324	cellular response to hormone stimulus
0042401	1.22 × 10^3^	12	50	cellular biogenic amine biosynthetic process
0071495	1.22 × 10^3^	49	352	cellular response to endogenous stimulus
0046148	1.36 × 10^3^	21	116	pigment biosynthetic process
0010033	1.52 × 10^3^	116	993	response to organic substance
0009723	1.55 × 10^3^	22	125	response to ethylene stimulus
0009608	1.65 × 10^3^	5	11	response to symbiont

**Table 5.** Over-represented GO terms of SF-specific DEGs.

GO-ID	*p*-value	Input number	Background number	Description
0016036	3.89 × 10^5^	8	52	cellular response to phosphate starvation
0000041	8.71 × 10^5^	9	74	transition metal ion transport
0007154	1.10 × 10^4^	15	195	cell communication
0009765	1.97 × 10^4^	8	65	photosynthesis, light harvesting
0009867	2.73 × 10^4^	6	37	jasmonic acid mediated signaling pathway
0071395	2.73 × 10^4^	6	37	cellular response to jasmonic acid stimulus
0006464	3.34 × 10^4^	75	2049	protein modification process
0035303	4.11 × 10^4^	4	15	regulation of dephosphorylation
0006950	4.38 × 10^4^	79	2205	response to stress
0050896	5.30 × 10^4^	119	3644	response to stimulus
0006664	5.55 × 10^4^	6	42	glycolipid metabolic process
0009875	5.57 × 10^4^	7	58	pollen-pistil interaction
0006827	6.15 × 10^4^	2	2	high-affinity iron ion transport
0046506	6.15 × 10^4^	2	2	sulfolipid biosynthetic process
0046505	6.15 × 10^4^	2	2	sulfolipid metabolic process
0006825	6.68 × 10^4^	5	29	copper ion transport
0009247	7.85 × 10^4^	5	30	glycolipid biosynthetic process
0009267	8.20 × 10^4^	8	80	cellular response to starvation
0009743	8.66 × 10^4^	12	165	response to carbohydrate stimulus
0043687	9.48 × 10^4^	68	1883	post-translational protein modification

**Table 6.** Over-represented GO terms of TF-specific DEGs.

GO-ID	*p*-value	Input number	Background number	Description
0015833	1.28 × 10^4^	6	50	peptide transport
0042454	1.29 × 10^4^	3	7	ribonucleoside catabolic process

**Table 7.** RNA-seq samples subjected to various treatments.

Cultivated regions	CF	SF	TF
KY	KYC	KYsTZ and KYsWN	KYP
TZ	TZC	TZsKY	TZP
WN	WNC	WNsKY	WNP

KYC, TZC, and WNC represent samples originating from KY, TZ, and WN without soil exchange and tillage treatment. KYsTZ and KYsWN represent samples harvested from KY grown in soils from TZ and WN, respectively. TZsKY and WNsKY indicate samples collected from TZ and WN and grown on KY soil. KYP, TZP, and WNP represent samples harvested from the corresponding cultivated regions with tillage treatment.
